# Event and Comment

**Published:** 1905-03-15

**Authors:** 


THE
### DENTAL REGISTER.

Vol. LIX. March 15, 1905. No. 3.

### EVENT AND COMMENT.

### Dentists Eat Too Much.

In another place of this issue we publish an article by an eminent New York physician which it would do well for some dentists at least to read carefully. In fact it will not be out of place for every dentist to read it carefully. The man under discussion was a Wall street broker, and undoubtedly led a feverish existence, and fell very naturally into the error of eating too much, and at a very early period in his life came near to incapacitating himself for his business, if not seriously threatening his existence. How many dentists who are carrying heavy practices take proper care to eat in such a manner as to best equip them for the strenuous life they are compelled to lead. A man who works hard must eat enough to replace the necessary lost energy. But do we eat the best food, and at the proper time, to best conserve our energies? The city dentist lives miles from his place of business. He eats a hearty breakfast and rushes for his train. He spends the time on the train reading the sensational newspapers, talking politics or smoking, and reaches his office with an undigested breakfast to handicap his morning's work, and at noon rushes to a nearby restaurant and satisfies his feverish temper, which he mistakes for an appetite, with a meal that a wood-chopper only could digest and rushes back to the office to find an acccumulation of patients each wishing to be served first, and there is no chance for a half hour's rest of mind and body that the digestion of the luncheon may get even fairly started before it is completely stopped by the unfavorable state of mind and an attitude which the work over the chair demands. Evening comes and the dinner is tempting and is taken in excess on top of two already only partially digested meals. An evening party, a late lunch and a troubled sleep follow to unfit us for another day of like experiences. Can we wonder then that we have indigestion, rheumatism, gout, pyorrhea and other troubles which really disqualify us for rendering our best services? When we think of this question of eating and the confinement in poorly ventilated offices it seems as though the dentist must be especially cared for by some good angel or he could not possibly live out half his natural life.

**Dr. W. D. Miller Honored.**

At the recent meeting of the Fourth International Dental Congress at St. Louis, Dr. W. D. Miller, of Berlin, was not only awarded the first prize for the best paper contributed to the Congress, but the following resolution was unanimously adopted as introduced by Dr. G. V. Black: *Resolved,* that the dental profession in the Fourth International Dental Congress assembled, wishes to express its gratitude to Dr. W. D. Miller, of Berlin, for the noble work done by him twenty years ago in developing the facts of the direct relation of bacteria to the causation of caries of the teeth, the truth of which has stood the test of many and repeated corroborations by others, and year by year has come to stand out more and more brilliantly as a great scientific achievement of lasting good to humanity.

**Age Limit of the Dental Practitioner.**

Considerable comment has been made on the recent alleged statement of Dr. Osler, the eminent teacher and physician of Johns Hopkins University, to the effect that men over the age of sixty years had no valuable function in the practical affairs of life, and the world would be no loser should they be chloroformed to rid the world of an incubus and to give younger and more vigorous men a chance to do the world's work. This is a suggestion that comes home to men in all walks of life; and naturally suggests the propriety of asking the question as to how long a dentist can practice his profession advantageously to the public. Of course every one will say at once that very much depends on the man, some men ought to quit before they begin, others ought to quit at forty-five when their eyesight begins to change, and others when they lose control of that steady hand which is of such great value in applying that delicate touch which most dental operations require. If it was possible for a dentist to lay by a sufficient of this world's goods to enable him to choose some less exacting employment for his old age, it might be practicable to set a limit for resigning practice; but the fact is that very few men at sixty years of age and after forty years of hard practice are able to discontinue practice because of financial ability. Most dentists have better than average incomes, yet few save much money. This is due to the fact that a dentist known to have a good income is expected, and practically forced, to take a position in society and community affairs which generally absorbs what surplus he ought to have above his necessary living expense, and it takes a long time to save enough to carry one in respectable living to the end of a life of seventy years, should he be forced to drop his profession at say sixty years. Consequently, we find dentists practicing their profession up to the limits of their physical capacities. It is not uncommon to find men in practice in the eighties. The dead line for preachers has been fixed at fifty; for physicians, by Dr. Osler, at sixty; we never heard of a lawyer being too old to take a case, and the dentist has not had a pronouncement of his doom as yet. The probabilities are that this question can not be arbitrarily answered. The public has a way of letting a man know when his services are not considered necessary, and as this process is perfectly natural and definite it does not seem worth while bothering ourselves about it. There is nothing like work to keep a boy out of mischief, and there is nothing like a congenial occupation to keep a man alive; and the sensible thing is for us to let the good Lord and our clients whom we are to serve with our whole beings adjust this matter for us, since it is altogether probable they will do this at the proper time. We can then silently slip away in the quiet of a conscience void of offense, or resort to the chloroform bottle, should it become necessary to quiet the ominous croaking of remorse for a life which has not been worth the living.

**Correspondence Schools.**

Almost every profession now has what are called correspondence schools, which are an outgrowth of the Chautauqua idea inaugurated several years ago as a compromise between a Methodist camp-meeting and a university. Because of the men who controlled the Chautauqua organization it has undoubtedly been an educational factor of great value to the country. As many persons who could not have attended a school of higher education have had outlined for them systematic courses of reading which when faithfully followed have resulted in materially increasing the general culture of the nation. The so-called correspondence schools, so long as they were confined to commercial affairs, have also done much for the increase of good business methods. The advent of these schools into professional training is a much more questionable matter. It might be possible to give a man a fair amount of training for the ministry, or the practice of law, by superintending at long distance a course of reading; but how it is going to be practicable to train a man for the practice of medicine through a course of correspondence instructions, which is now being advertised, is more than we can see. It certainly is contrary to our present conceptions of an ideal medical training. A man might read about measles, small-pox and similar diseases until he was too old to attempt to treat a case and still be utterly unfit to make a differential diagnosis when called to treat such cases. No correspondence school for dentistry has as yet appeared, but it will probably not be long before some get-rich quick young man will be announcing one. Some of our dental journals are running in their current volumes matter which could easily be utilized and perhaps they are unconsciously "blazing the way" for this sort of an enterprise. If it can be done in medicine, it surely can in dentistry, as the technical department of dentistry is capable of being clearly demonstrated by pictures, drawings and models. The prevailing laws for licensing practitioners may serve to prevent any considerable exploitation of this method. However, we have heard of a young man who never attended a dental college, nor was ever even in a dental office except on commercial business, or for professional services, who took the text-books and prepared himself by reading so well that he took and passed the State Board examination of one of our largest States.

**Fees.**

There is perhaps no subject which furnishes more opportunities for discussion in dental societies than the question of fees. It is quite important that there should be some understanding, especially in small communities, as to the minimum fee for certain services which are relatively common and frequently required. Such, for instance, as extracting a tooth or making a gold crown or a rubber plate, etc. But this particular feature is not the one we wish to call attention to here. It is often difficult for a young and conscientious practitioner to determine the value of an unusual operation, which he has skillfully made and which has cost him more than ordinary effort to accomplish satisfactorily to his patient. Generally such a dilemma is adjusted from the patient's standpoint rather than the operator's. If the patient is willing and able to pay a fee which is satisfactory to the operator the problem is simple; but if the patient is able and unwilling it becomes complex or may be at least unpleasant. Should the patient be both unwilling and unable there seems to be absolutely no hope of a satisfactory solution except to credit the extra effort to benevolence or "treasures in heaven," which is good enough when it doesn't come so often as to create a spirit of rebellion. The adjustment of the ordinary fees and these special ones may be taken care of as they come along; but the ordinary operations and the average fees are not sufficiently compensatory for the man who puts his whole energy of every sort into them. The usual price for extracting a tooth may be fifty cents, and ordinarily the dentist is satisfied with such a fee, because he extracts the tooth on the fifty cents basis. But if he uses five dollars' worth of time, judgment and skill, is he doing himself justice in accepting a fifty cent fee? We are so accustomed to follow traditions that we fail to cultivate individuality. There can be no incentive that comes naturally to a man from conducting his business on such principles. If he is willing to accept traditional fees the incentive to render superior service must be engendered by pure will power and can never be spontaneous, or result in an aspiration to do better things than others. It will require only a little observation to convince any one of this fact. The men who are doing things out of the ordinary are getting better fees for their services than the ordinary practitioners. Patients will pay larger fees to the man who puts his very life into his operations. Of course there are lots of patients who are built on the narrow plan, but patients that are doing things themselves recognize as a kindred spirit professional men who love their work and follow it with all the character they have or can by any means cultivate. The moral then is to put one's whole ability in every operation, however trifling it may seem, and ask such a fee for the service as shall inspire to greater efforts for increased capacity.

**Dental Literature.**

This is the season when we are asked by the publishers to subscribe for the new year. We never see these notices on the covers of our exchanges that we are not reminded of the oft-repeated statement that we are not a reading profession. It seems strange that such a statement can be made without contradiction, especially in this day, when we have so many really good things to read that ought to be read and pondered and practiced. There never was a time when the dental periodical literature was so good and inexpensive as it is to-day. The dental journals contain the best thoughts of the best workers in the country, and there is not one journal printed that will not more than repay its cost if carefully read every year. The better journals contain in a single number more than enough to pay the cost of the entire subscription. The most widely-circulated journal does not reach one-third of the practitioners of this country. Not more than one-third of the profession take more than three journals, when a five-dollar bill will bring to them each month five of the best journals the world has ever known. Fifteen dollars will buy every journal of consequence published in the United States. We are not writing this in behalf of the publishers, but for the sake of our profession. For our own personal advancement we must read our own literature. If we read carefully we will do what others are doing at least, and we may be inspired to do things that no one else has done. Do things and write them up and send them to your journals that others may imitate your work. Medical men have a good habit that dentists ought to imitate, and that is to relate cases in practice; there is nothing so interesting or likely to inspire others to make their works known.

**A Protest by Berlin American Dentists.**

At the time of the death of Dr. Sylvester in Berlin, which occurred recently by suicide, the *German Times*, a newspaper published in the English language in Berlin, stated that one direct cause for the suicide was due to the dissolution of the partnership which had existed between Dr. Sylvester and Dr. G. H. Watson. The newspaper intimated that Dr. Watson had taken advantage of Dr. Sylvester in the settlement, to Sylvester's financial injury. We are glad to present herewith a signed statement from the American dentists of Berlin which speaks for itself:

"We, the undersigned American dentists in Berlin, respectfully present the following urgent protest against a notice in the last number of the *German Times* (January 16th), in which statements are made which are well calculated to injure the name of a member of the American colony who is most highly esteemed not only as a skillful practitioner of dentistry, but as a man of unwavering integrity of character. Having examined into the business relations of this gentlemen with the deceased Dr. Sylvester, we are absolutely convinced that he not only most conscientiously and scrupulously lived up to the terms of the contract which existed between him and the deceased, but still more that he evinced a charity in his dealings far beyond that which could have been legally exacted and which has evoked our undivided admiration.

"We herewith express our unreserved confidence in the gentleman in question and our strong disapproval of all attempts to make him in any way responsible for a deplorable event over which he had no control."

Signed:

W. D. MILLER, GEORGE MARTIN, GEO. A. KENNEDY,
FERD. FOERSTER, J. H. RAMSEY, LEE A. WATLING,
CHAS. H. ABBOT, E. LAWLEY-YORK. E. D. BARROWS.
GEO. O. WEBSTER,

BERLIN, January 19th, 1905.

**Annual Report.**

The Wisconsin State Board of Examiners has issued in printed form its annual report to the Governor of the State. The report contains not only the Board's transactions, but the State law and a roster of all the names of the registered practitioners of the State, with their addresses. The report also prints a list of about seventy names of such persons as have not paid their annual re-license tax of one dollar. It seems that the Board notifies each registered practitioner when his license expires and should he fail to have it renewed in ninety days his license shall be revoked and he must take a new examination before the Board will re-license him. This is not a bad method of keeping the rolls of practitioners cleared up, but it seems a great deal of a nuisance to have to register every year. The question naturally arises to one's mind as to whether such a law is constitutional, in view of the fact that many State Boards decline to issue temporary licenses, because of the fact that such a license would be considered good evidence that if such a person was qualified for a temporary license he was also qualified to practice permanently. We don't know that this point has ever been legally tested, but it would furnish good testimony to a lawyer who was trying to break down a State law containing such a clause.

